# Supplementary material for: How Nanoparticles Modify Adsorbed Proteins: Impact of Silica Nanoparticles on the Hemoglobin Active Site
Source: Int J Mol Sci. 2023 Feb 11;24(4):3659. doi: 10.3390/ijms24043659 (PMC9967434; doi:10.3390/ijms24043659)
Supplement: Supplementary file 1 [file ijms-24-03659-s001.zip › ijms-2194151-supplementary.pdf]

Supporting Information

How nanoparticles modify adsorbed proteins: Impact of silica nanoparticles on the hemoglobin active site

Gaël Giraudon--Colas[1], Stéphanie Devineau[2], Laurent Marichal[1], Elodie Barruet[1], Andrea Zitolo[3], Jean Philippe Renault[1]*, Serge Pin[1]

[1] Université Paris-Saclay, CEA, CNRS, NIMBE, 91190-Gif-sur-Yvette, France

[2] Université Paris Cité, CNRS, Unité de Biologie Fonctionnelle et Adaptative, F-75013 Paris, France

[3] Synchrotron SOLEIL, L'Orme des Merisiers, BP 48 Saint Aubin, 91192 Gif-sur-Yvette, France

Figure S1

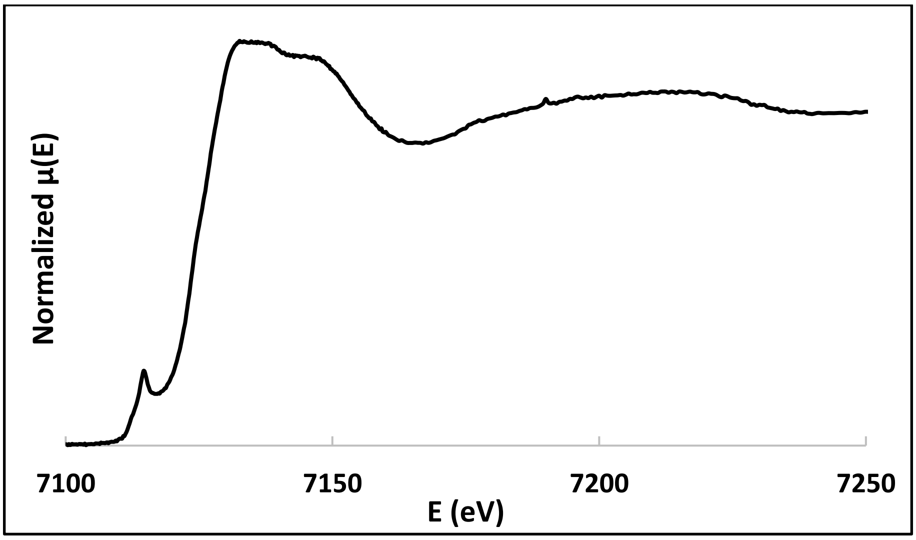

Figure S1. Normalized Fe K-edge XANES spectra of silica nanoparticles containing Fe impurities. Data recorded for a NP concentration of 120 g.L^{-1} in 0.1 mol.L^{-1} phosphate pH 7.0 at 20 K.

The XANES spectra of NPs strongly resembles that already studied in disordered mesoporous silica that has been functionalized with iron atoms, which was interpreted as iron III in a tetrahedral environment [1].

Table S1. Pre-edge and threshold positions (E_0) of Fe K-edge XANES spectra of 10 mM oxyhemoglobin, 10 mM deoxyhemoglobin and 120 g.L^{-1}silica nanoparticles containing Fe impurities. Data recorded in 0.1 mol.L-1 phosphate pH 7.0 at 20 K.

Energy (eV)	Pre-edge oxyHb	Pre-edge deoxyHb	Pre-edge NP	E_0 oxyHb	E_0 deoxyHb	E_0 NP
	7113.6	7113.0	7114.6	7122.2	7118.4	7124.0

Table S2. Iron proportions for the adsorbed conditions (98% adsorbed) used for the XAS experiments: 1.0 mM Hb and 120 g.L^{-1} NP in 0.1 mol.L^{-1} phosphate pH 7.0.

	C(Fe) in mM				% Fe		
	Free Hb	Adsorbed Hb	Ludox NPs		Free Hb	Adsorbed Hb	Ludox NPs
Ludox NPs	0,02	0,98	0,55		1,3	63,3	35,4

Figure S2

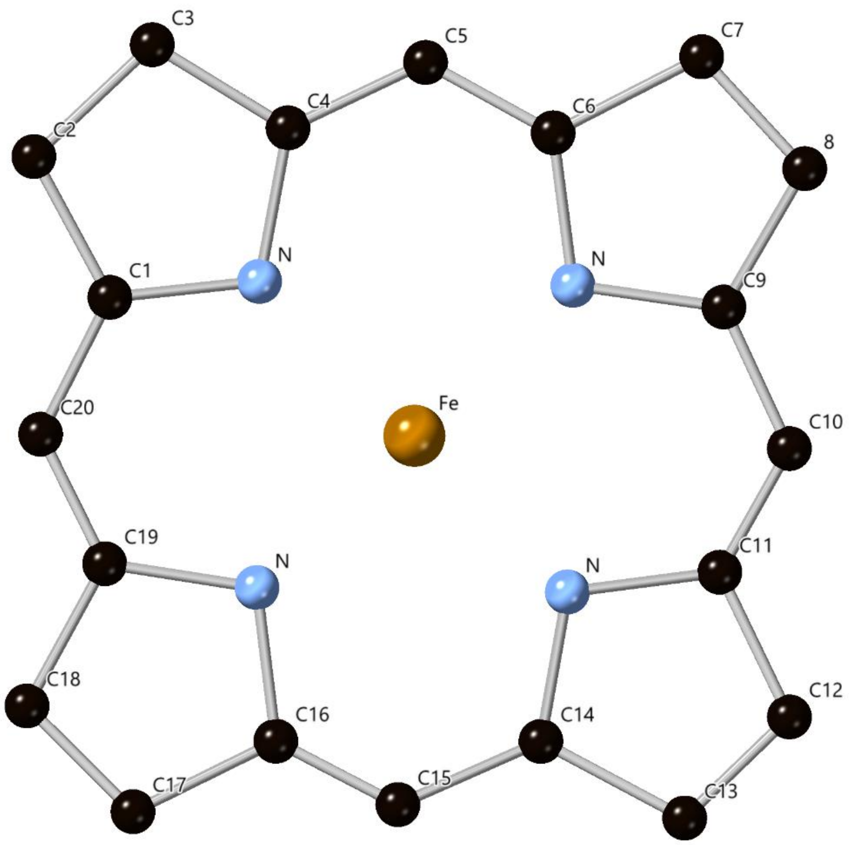

Figure S2. Sketch of the porphyrin macrocycle with the standard numbering scheme.
Carbon atoms at positions 5, 10, 15 and 20 are referred to as the meso carbons.
Carbon atoms at 1, 4, 6, 9, 11, 14, 16 and 19 are the Ca carbons
while the Cb carbons are at 2, 3, 7, 8, 12, 13, 17 and 18 positions.

Figure S3

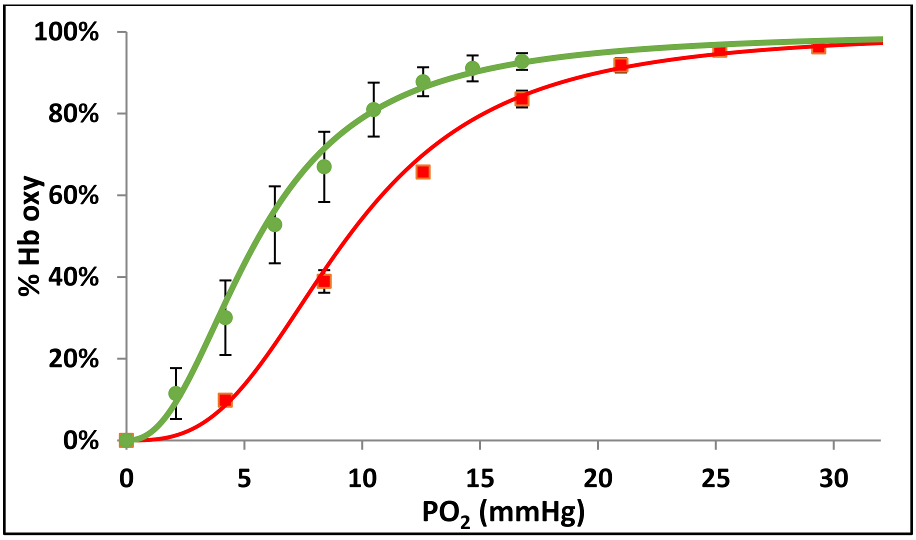

Figure S3. Oxygen binding curves of adsorbed (green circles,) and free (red squares) hemoglobin.
The solid lines show the fits obtained with the Hill model.
Data were recorded with 63 µM Hb in 0.1 mol.L^{-1} phosphate pH 7.4 at 25°C for free Hb and with 63 µM Hb and a NPs concentration of 38.4 g .L^{-1} in 0.1 mol.L^{-1} phosphate pH 7.4 at 25°C, more than 95% Hb were absorbed.

Table S3. O_2 dissociation constants K_1, K_2, K_3, K_4 and free enthalpy Δ_rG^0 at 25°C for adsorbed and free porcine hemoglobin (Hb) in 0.1 mol.L^{-1} phosphate pH 7.4. Values obtained from the Adair model applied to the oxygen binding curves shown in Figure S3. The data for human Hb come from Gill et al. [2] for phosphate buffer pH 7.5 at 25 °C.

	Free porcine Hb	Absorbed porcine Hb		Free human Hb
K_1 (Torr^{-1})	8.5×10^{-2}	1.9×10^{-1}		3.9×10^{-2}
K_2 (Torr^{-2})	1.7×10^{-3}	1.9×10^{-3}		6.3×10^{-3}
K_3 (Torr^{-3})	5.3×10^{-5}	3.0×10^{-3}		0
K_4 (Torr^{-4})	1.4×10^{-4}	9.1×10^{-4}		1.7×10^{-4}
Δ_rG^0 (kJ.mol^{-1})	-108.5	-113		

Scheme S1. The values presented come from Table 1 and Table S3

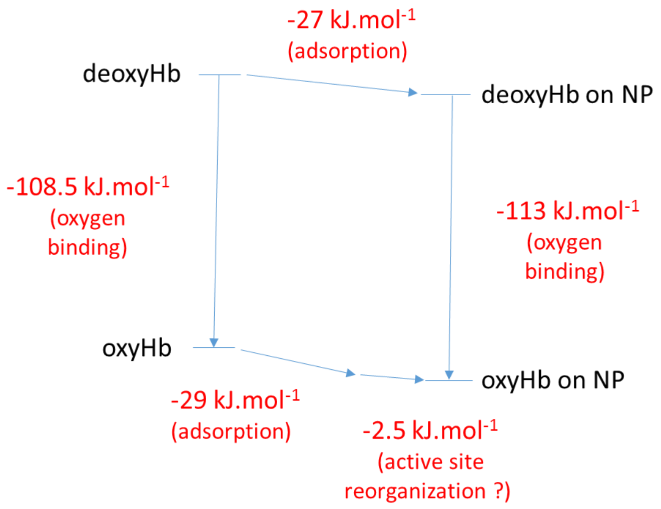

Figure S4

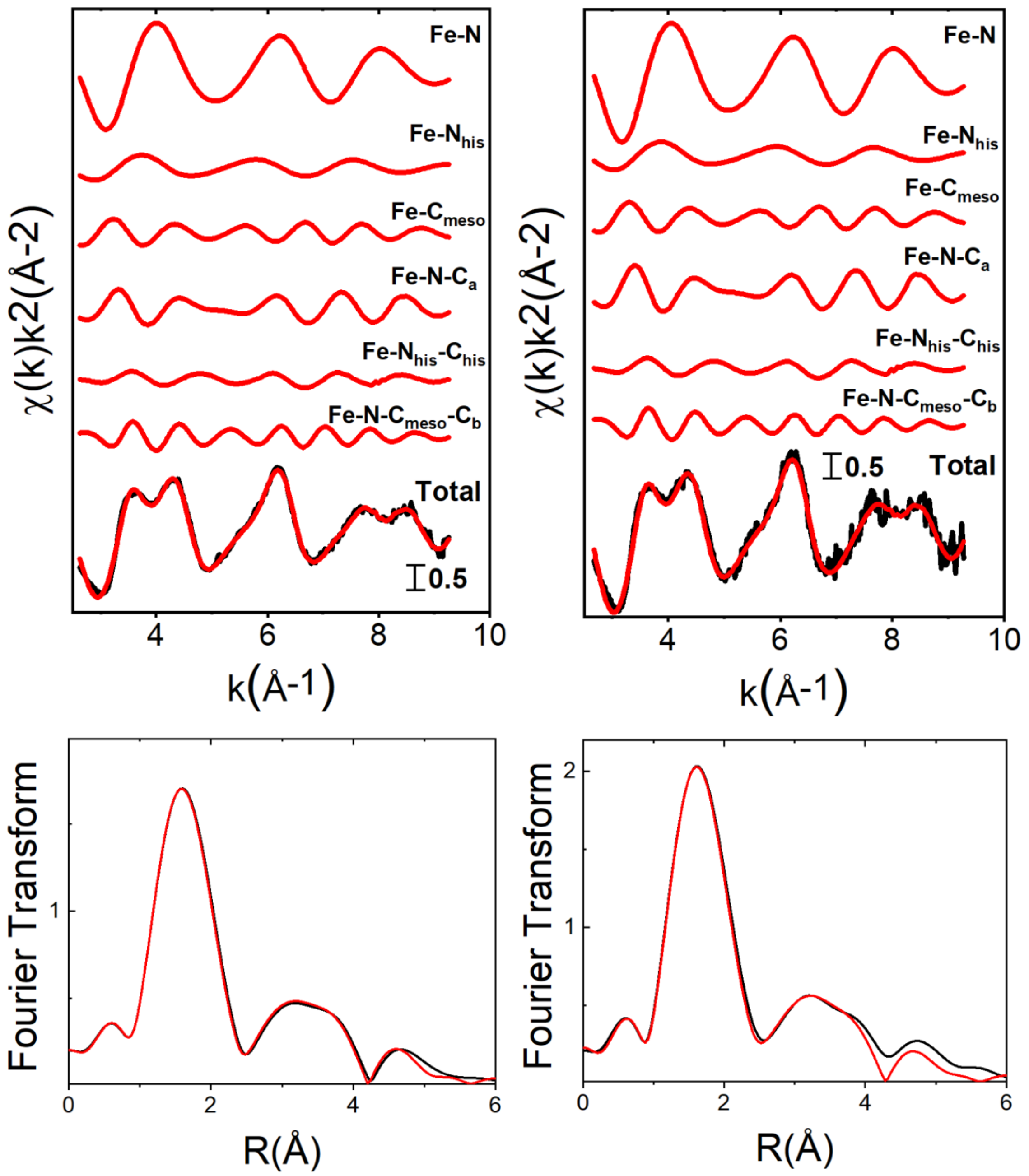

Figure S4. Fe K-edge EXAFS analysis of the free and adsorbed deoxyHb.
Left panel, free deoxyHb: from top to bottom
two-, three-, and four-body contributions included in the fit (red curves),
the total theoretical signal (red curve) superimposed to the experimental one (black curve),
and the fit in the Fourier transformed space.
Right panel, the same analysis for adsorbed deoxyHb.

Table S4. First shell best-fit parameters obtained from the Fe K-edge EXAFS analysis of oxyHb and deoxyHb in the free and adsorbed forms. R is the interatomic distance, σ^2 represents the Debye-Waller factor. Errors are given in parentheses, e.g., 2.02(1) means 2.01-2.03.

		R(Å)	σ^2 (10^{-3} Å^2)
oxyHb free	Fe-N	2.02(1)	1.5(4)
	Fe-O	1.85(2)	1.0(1.0)
	Fe-N_{his}	2.26(8)	2.0(6)
deoxyHb free	Fe-N	2.05(1)	2.6(4)
	Fe-N_{his}	2.17(5)	1.8(7)
oxyHb adsorbed	Fe-N	2.01(1)	1.5(5)
	Fe-O	1.86(3)	1.0(1.0)
	Fe-N_{his}	2.23(7)	1.9(5)
deoxyHb adsorbed	Fe-N	2.06(2)	1.3(6)
	Fe-N_{his}	2.15(3)	1.0(6)

References

[1] Rangus, M.; Mazaj, M.; Dražić, G.; Popova, M.; Tušar, N. N. Active Iron Sites of Disordered Mesoporous Silica Catalyst FeKIL-2 in the Oxidation of Volatile Organic Compounds (VOC). Materials 2014, 7 (6), 4243–4257.

[2] Gill, S.J; Di Cera, E.; Doyle, M.L.; Bishop, G.A.; Robert, C.H., Oxygen binding constants for human hemoglobin tetramers. Biochemistry 1987, 26 (13) 3995–4002
